# Dr. S. C. Hitchcock

**Published:** 1871-06

**Authors:** 


### DR. S. C. HITCHCOCK.

We beg to tender our best thanks to the above-named gentleman for his kindness in presenting the editors with a box of his soap. Dr. Hitchcock is an old practitioner, having by his profession, secured a handsome competency. He is now engaged in this city in the manufacture of a superior quality of soap. We have had his soap practically tested, and we unhesitatingly say it is inferior to none in the market—equalling Babbitt's best brand—besides being much cheaper as to price. The Doctor is a gentleman worthy of the esteem and patronage of all, and those dealing with him will find him obliging and strictly honorable in his transactions. We ask our friends who want a first-rate article of soap to give the Doctor a trial—they will not be disappointed.

